# Use of analgesics before and after total joint replacement in working-age Japanese patients with knee and hip osteoarthritis: A retrospective database study

**DOI:** 10.1016/j.asmart.2023.10.002

**Published:** 2023-11-03

**Authors:** Nozomi Ebata, Takashi Sakai, Hiroyuki Yamamoto, Tetsumi Toyoda, Kanae Togo, Masataka Deie

**Affiliations:** a Pfizer Japan Inc., 3-22-7 Yoyogi, Shibuya-ku, Tokyo, 151-8589, Japan; b Department of Orthopedic Surgery, Yamaguchi University Graduate School of Medicine, 1-1-1 Minami-Kogushi, Ube, Yamaguchi, 755-8505, Japan; c Clinical Study Support, Inc., 1-11-20 Nishiki, Naka-ku, Nagoya, Aichi, 460-0003, Japan; d Department of Orthopaedic Surgery, Aichi Medical University, 1-1 Yazago-Karimata, Nagakute, Aichi, 480-1195, Japan

**Keywords:** Analgesics, Hip osteoarthritis, Knee osteoarthritis, Non-steroidal anti-inflammatory drugs, Opioids, Total joint replacement

## Abstract

**Background:**

Patterns of analgesic use before and after total joint replacement (TJR) in patients with knee/hip osteoarthritis (OA) is not well reported.

**Methods:**

This retrospective longitudinal analysis used JMDC claims data of patients who underwent knee/hip replacement surgery from 2010 to 2019. Primary outcome was proportion of patients using analgesics pre-surgery, immediately post-surgery, and in post-surgery period. Factors affecting post-surgery analgesic withdrawal and opioid prescriptions were assessed using logistic regression.

**Results:**

Of all (N = 3168) patients, those with knee OA (91.1 %) and hip OA (82.5 %) used analgesics pre-surgery, and 96.1 % with knee OA and 84.9 % with hip OA required analgesics even 3 months post-surgery. NSAIDs were most commonly used pre- and post-surgery in both OA groups. Before surgery, 15.6 % (knee OA) and 13.7 % of patients (hip OA) used weak opioids, and 23.1 % (knee OA) and 10.5 % (hip OA) of patients continued them post-surgery. Strong opioid use was noted in 2.2 % and 1.2 % of patients pre-surgery, and 5.8 % and 3.4 % of patients post-surgery in the knee and hip OA groups, respectively. Using pre-operative oral NSAIDs (odds ratio [OR]:0.56; 95 % confidence interval [CI]:0.44–0.72) and weak opioids (OR:0.58; 95 % CI:0.38–0.87) associated with withdrawal of post-surgery analgesics in patients with hip OA, and using intra-articular hyaluronic acid pre-surgery (OR:0.45; 95 % CI:0.21–0.97) was significant in patients with knee OA. Using weak (OR:4.59; 95 % CI:3.44–6.13) and strong opioids (OR:2.48; 95 % CI:1.01–6.07) pre-surgery associated with post-operative opioid use in patients with hip OA, and weak opioid use was significant in patients with knee OA (OR:7.00; 95 % CI:4.65–10.54).

**Conclusion:**

This study reported difference in analgesic use before and after TJR, and that many patients required analgesics even 3 months after TJR surgery in Japan. Pre-operative analgesic use associated with continued use after surgery. Optimal pain management before and immediately after TJR is important to reduce post-operative analgesic use, especially opioids.

## 1 Introduction

Osteoarthritis (OA), the most common type of arthritis, reportedly affected >300 million people worldwide in 2017.^1^ Pain is a key symptom that prompts patients with OA to seek medical care, and is an important preceding sign to disability.^2^^,^^3^ Managing OA involves a multidisciplinary approach including pharmacological, non-pharmacological, and surgical therapies that aim at relieving pain using analgesics, and improving joint function and quality of life (QoL).^4^^,^^5^ Clinical practice guidelines recommend total joint replacement (TJR) in patients who continue to experience pain despite conservative therapies.^6^ However, a significant proportion of patients, approximately 7%–23 % of patients after a total hip replacement (THR) and 10%–34 % of patients after total knee replacement (TKR), continue to experience post-operative pain.^7^

A few studies have evaluated differences in medications, health care resource use, and costs before and after total knee/hip arthroplasty in patients with OA.^8^^,^^9^ A recent study in Finland reported increased use of analgesics prior joint replacement, which reduced post-surgery analgesic use. However, a considerable proportion of patients (23 % of hip replacement and 30 % of knee replacement patients) continued using analgesics to manage OA-related pain.^10^ Increased age, multiple comorbidities, obesity, and pre-operative analgesic use were the strongest predictors of increased post-operative analgesic use in this setting.^10^

Prevalence of OA is rising in Japan due to aging, so is the use of analgesics and TJR for pain relief.^11^ A few studies showed that pre-operative analgesic use, and other factors associated with their post-operative use that was possibly due to uncontrolled, prolonged post-operative pain after TJR.^10^^,^^12^^,^^13^ Data regarding difference in using analgesic before and after TJR surgery in patients with knee/hip OA, and the factors that affect their prescription after TJR, are not available in Japan. Our study compared pre- and post-operative analgesic-use in patients with knee/hip OA in Japan who underwent knee/hip TJR. Pre-surgical factors influencing prescription of analgesics post-surgery (including opioids) were also assessed.

In this database analysis, we hypothesized that TJR would reduce pain after surgery and consequently reduce analgesic use after surgery.

## 2 Methods

### 2.1 Study design and data sources

This was a retrospective, longitudinal, observational cohort study that used claims data from the JMDC database (formerly named as Japan Medical Data Center Co.,Ltd., largest claims database commercially available in Japan) that contains all claims (data of ∼9.6 million patients) across multiple medical institutions who received inpatient and outpatient treatment, and pharmacy claims made by Japanese health insurance companies for employees and their family members who are <75 years old.^14^ The JMDC database codes diseases according to the Japanese Claims Codes and the coding scheme of the World Health Organization International Classification of Diseases, 10th revision (ICD-10).

Patients who had undergone knee/hip replacement surgery, were ≥18 years at the index date with at least one record of ICD-10 diagnosis of knee or hip OA (ICD-10 codes M16.X and M17.X) before the index date, were included. These patients were followed-up for up to 3 months before and after surgery, and had ≥2 prescriptions (with ≥1-month gap) for analgesic-use after the initial diagnosis of OA. The study observation period was from January 1, 2010, to December 31, 2019. Index date was the date when TJR was performed. Analgesics included oral and non-oral non-steroidal anti-inflammatory drugs (NSAIDs), acetaminophen, intra-articular hyaluronic acid and steroid injection, weak (tramadol, codeine, and buprenorphine) and strong opioids (fentanyl, morphine), duloxetine, and vaccinia virus-inoculated domestic rabbit inflammatory skin extract. Analgesics not indicated in Japan for OA were excluded. Key exclusion criteria included patients with THR/TKR during both pre-surgery (from index date −91 days to index date −1 day) and post-surgery periods (from index date +7 days to index date +89 days), and those with ICD-10 codes of malignancy [C00–C97, D00-D09], and rheumatoid arthritis [M05.X, M06.X] during the observation period.

The present study involved use of data within a pre-existing database, and primary data collection was not performed. The Japanese Ethical Guidelines “Medical and Health Research Involving Human Subjects” do not apply to studies that use anonymized secondary data (Part 3 “Scope of Application” on page 10); therefore, approval from an institutional review board/research ethics committee was deemed not necessary. No informed consent was sought from patients.

However, the study was conducted in accordance with the Guidelines for Good Pharmacoepidemiology Practices (GPP) issued by the International Society for Pharmacoepidemiology (ISPE). The study was reported in compliance with the REporting of studies Conducted using Observational Routinely-collected health Data (RECORD) statement.

The primary outcome of the study was the proportion of patients using analgesics before and after surgery, defined as drug use during the pre-surgery period, immediate post-surgery period (from index date to index date +6 days), and post-surgery period. Secondary outcomes included factors affecting withdrawal of analgesics drugs during the post-surgery period and those affecting opioids prescription during the post-surgery period. These outcomes were calculated separately for patients with TKR and those with THR. The percentage of prescription for analgesics in patients discharged from facilities where TJR was performed but who visited other facilities post-surgery were also calculated. This analysis was performed assuming that fewer analgesics would be prescribed when patients would visit other facilities after surgery.

### 2.2 Statistical analysis

All patients who met the eligibility criteria were included in the analysis set. For categorical data, frequency and percentages of patients were summarized. For continuous variables, mean, standard deviation (SD), median, and interquartile range (IQR) were calculated. The percentage of medication used before and after TJR were summarized with their 95 % confidence interval (CI). Two separate multivariate logistic regression analyses were performed: one was to identify the factors affecting the prescription of analgesics post-operatively for hip and knee OA patients separately. The other was to identify the factors affecting the opioids prescription post-operatively for hip and knee OA patients separately. The model variables included patients’ characteristics: age, gender, comorbidities, and pre-surgery treatment. An alpha level of 0.05 was considered statistically significant. Analysis was performed using SAS Release version 9.4 (SAS Institute, Inc., Cary, NC, USA).

## 3 Results

### 3.1 Patient disposition

**Fig. 1:**
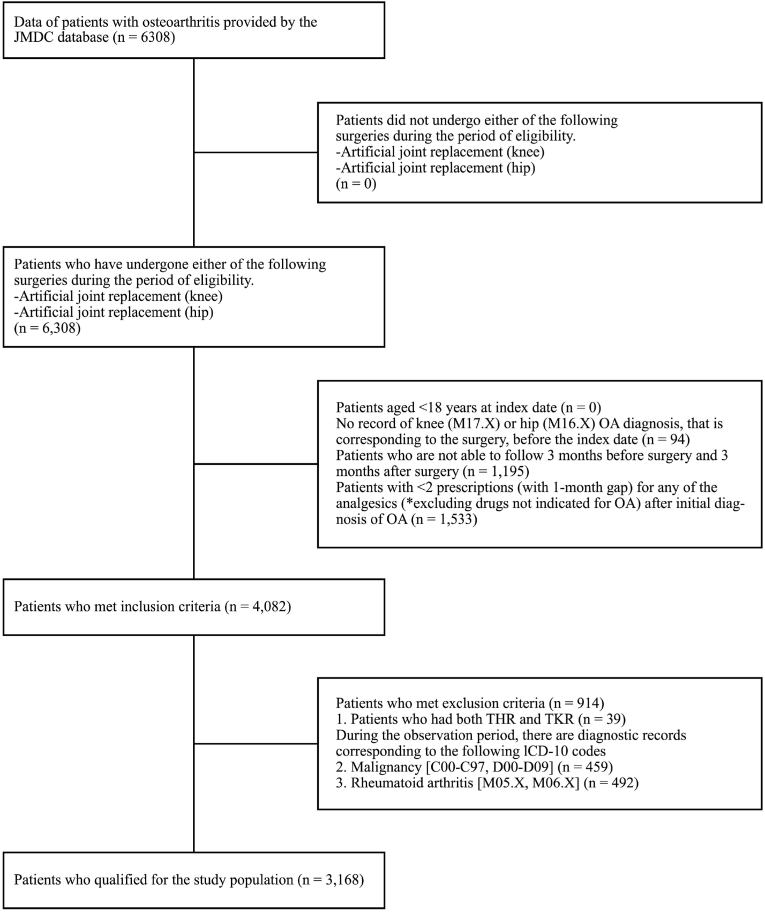
Patient disposition.

Of the 6308 patients who underwent TJR of the knee/hip between January 2010 and December 2019, 4082 patients met the inclusion criteria. After excluding 914 patients who had THR or TKR or a record of malignancy or rheumatoid arthritis during the observation period, 3,168 patients were included in the final analysis (Fig. 1).

### 3.2 Patient characteristics

Patient demographics and clinical characteristics are described in Table 1. Mean ± SD age of patients was 63.9 ± 6.60 years and 57.2 ± 7.32 years for TKR and THR, respectively. The proportion of elderly patients (≥65 years) was greater in the TKR group than in the THR group. The majority of patients were women in both the groups (68.3 % and 78.6 %, respectively). The most common comorbidity in patients with TKR was essential hypertension (50.1 %) followed by disorders of lipoprotein metabolism and other lipidemia (35.2 %) and mental disorders (29.6 %). While common comorbidities in THR patients were iron deficiency anemia (44.4 %) followed by essential hypertension (26.1 %) and mental disorders (25.5 %). Median (IQR) time from the first diagnosis of OA was 671.0 (304.0, 1248.0) days in patients with TKR and 466.0 (204.0, 969.0) days in patients with THR.

**Table 1:** Patient demographic characteristics.

Parameter	Knee OA N = 890	Hip OA N = 2278
Age, years		
Mean	63.9 ± 6.60	57.2 ± 7.32
Median	64.0 (60.0, 69.0)	57.0 (53.0, 62.0)
Age category (years), n (%)		
<55	67 (7.5)	769 (33.8)
55-64	404 (45.4)	1184 (52.0)
≥65	419 (47.1)	325 (14.3)
Gender, n (%)		
Male	282 (31.7)	487 (21.4)
Female	608 (68.3)	1791 (78.6)
Comorbidities, n (%)		
Essential (primary) hypertension	446 (50.1)	594 (26.1)
Disorders of lipoprotein metabolism and other lipidemia	313 (35.2)	430 (18.9)
Sleep disorders	255 (28.7)	509 (22.3)
Gastritis and duodenitis	246 (27.6)	476 (20.9)
Other functional intestinal disorders	225 (25.3)	408 (17.9)
Spondylosis	158 (17.8)	412 (18.1)
Iron deficiency anaemia	222 (24.9)	1011 (44.4)
Phlebitis and thrombophlebitis	171 (19.2)	377 (16.5)
Mental disorders	263 (29.6)	582 (25.5)
Gastro-esophageal reflux disease	208 (23.4)	–
Unspecified diabetes mellitus	182 (20.4)	–
Dorsalgia	–	371 (16.3)
Presence of other functional implants	–	344 (15.1)
Duration of OA, days		
Mean	889.7 ± 758.98	689.3 ± 662.83
Median	671.0 (304.0, 1248.0)	466.0 (204.0, 969.0)
Duration category, n (%)		
<365 days	266 (29.9)	957 (42.0)
≥365 days	624 (70.1)	1,321 (58.0)
Duration of OA (in patients who were prescribed opioids post-operatively), days		
Mean	923.1 ± 788.43	661.9 ± 631.53
Median	663.0 (314.5,1291.0)	446.0 (197.0,944.0)
Duration category (in patients who were prescribed opioids post-operatively), n (%)		
<365 days	67 (27.5)	128 (42.8)
≥365 days	177 (72.5)	171 (57.2)
Number of drugs used per patient (oral and non-oral NSAIDs counted separately), mean±SD		
Pre-surgery period	1.9 ± 1.11	1.2 ± 0.81
Immediate post-surgery period	2.7 ± 1.03	2.5 ± 0.93
Post-surgery period	1.7 ± 0.92	1.2 ± 0.84
Number of drugs used per patient (oral and non-oral NSAIDs counted as one class/drug)		
Pre-surgery period	2.3 ± 1.35	1.5 ± 1.04
Immediate post-surgery period	3.2 ± 1.17	2.9 ± 1.10
Post-surgery period	2.2 ± 1.13	1.5 ± 1.07

*NSAID* non-steroidal anti-inflammatory drug, *OA* osteoarthritis.

Comorbidities were defined as per the ICD-10 classifications (first 3 digits) ranked from 1st to 10th and mental disorder as comorbidity of interest (in descending order of the knee OA group). Proportion of comorbidities was calculated with number of patients in target population as denominator and number of patients with target disease as numerator.

### 3.3 Prescription of analgesics

As shown in Fig. 2A, 91.1 % and 82.5 % of patients were using analgesics before surgery, and 96.1 % and 84.9 % of patients continued using them in the post-surgery period in knee OA and hip OA groups, respectively. During pre-surgery and post-surgery periods, NSAIDs were the most commonly used analgesics, while in the immediate post-surgery period, opioids were the most common analgesics for both knee and hip OA (Fig. 2B, Fig. 2CB and C). Use of non-oral NSAIDs was higher in patients with knee OA compared to hip OA before TJR. Acetaminophen was used by 9.6 % and 9.1 % of patients before surgery, and these values increased post-surgery (18.9 % and 13.3 %) in the knee and hip OA groups, respectively. Intra-articular steroids were used by 21.3 % and 6.6 % of patients before surgery, and these reduced to 7.9 % and 5.9 % in the post-surgery period in the knee and hip OA groups, respectively. The proportion of patients who used weak opioids were 15.6 % and 13.7 % before surgery, and the values increased to 23.1 % in the knee OA and reduced to 10.5 % in the hip OA group. Similarly, strong opioid use was seen in 2.2 % and 1.2 % of patients and remained at 5.8 % and 3.4 % in patients with knee and hip OA, respectively. The use of intra-articular hyaluronic acid reduced after surgery. The proportion of patients using antidepressants before surgery was 2.9 % and 1.8 % in the knee and hip OA groups, respectively; these values increased in the post-surgery period. Furthermore, 356 (40 %) and 677 (29.7 %) of patients, respectively, with knee and hip OA were discharged from the facilities where the TJR was performed but later visited other facilities for pain. In these patients, as shown in Table S1, NSAIDs were the most prescribed analgesics (knee: oral 55.1 %, non-oral 61.2 %; hip: oral 52.7 %, non-oral 48.0 %).

**Fig. 2A:**
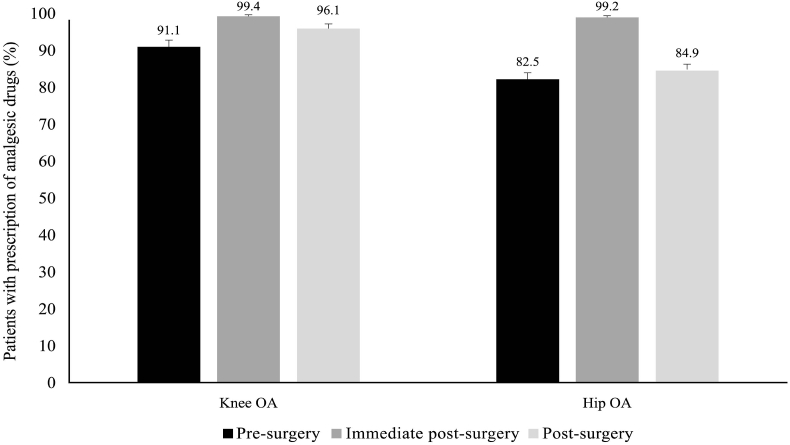
Proportion of patients prescribed analgesics in pre-surgery, immediate post-surgery, and post-surgery periods. Error bars represent 95 % CI.

**Fig. 2B:**
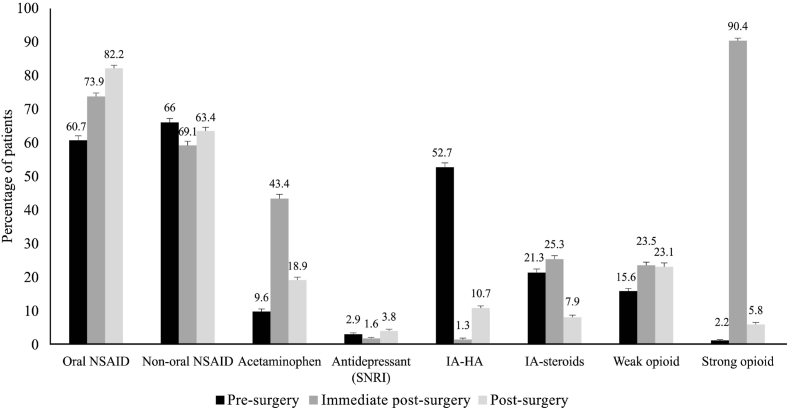
Analgesic use in patients with knee osteoarthritis. Error bars represent 95 % CI.

**Fig. 2C:**
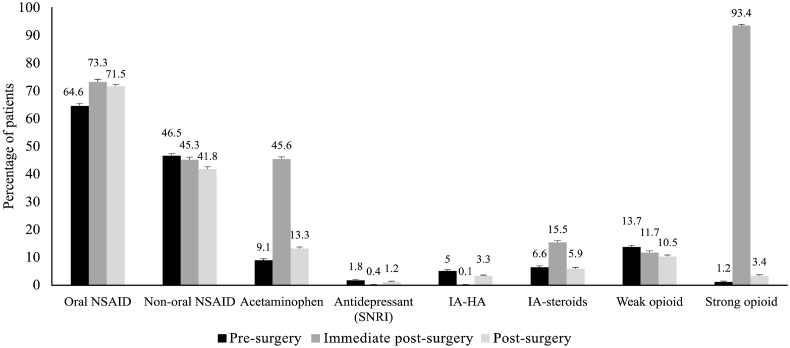
Analgesic use in patients with hip osteoarthritis. Error bars represent 95 % CI.

The aforementioned results corroborated with the mean number of analgesics used by patients as the number of analgesics used per patient increased in the immediate post-surgery period compared to that before surgery in the knee and hip OA groups (Table 1). Moreover, NSAIDs, opioids, antidepressants, and extract from inflamed cutaneous tissue of rabbits inoculated with vaccinia virus were used on most days in pre-surgery, immediately post-surgery, and post-surgery periods as shown by median prescription days (Table 2).

**Table 2:** Prescription (pain medications) days.

Parameter	Knee OA N = 890			Hip OA N = 2278		
	**Pre-surgery period**	**Immediate post-surgery period**	**Post-surgery period**	**Pre-surgery period**	**Immediate post-surgery period**	**Post-surgery period**
NSAIDs (oral), mean±SD	45.4 ± 36.89	9.5 ± 6.28	48.3 ± 35.50	51.2 ± 37.92	9.5 ± 6.87	36.7 ± 32.96
Median (IQR)	35.0 (14.0, 70.0)	7.0 (6.0, 12.0)	40.0 (21.0, 70.0)	43.0 (20.0, 80.0)	7.0 (6.0, 11.0)	28.0 (14.0, 50.0)
NSAIDs (non-oral)	3.5 ± 3.55	2.2 ± 1.77	3.9 ± 4.77	2.8 ± 2.58	1.9 ± 1.45	2.6 ± 2.68
Median (IQR)	3.0 (1.0, 5.0)	2.0 (1.0, 3.0)	3.0 (1.0, 5.0)	2.0 (1.0, 3.0)	1.0 (1.0, 2.0)	2.0 (1.0, 3.0)
Acetaminophen	23.7 ± 33.09	4.5 ± 6.85	31.0 ± 34.74	17.9 ± 28.03	3.9 ± 5.80	20.3 ± 24.66
Median (IQR)	7.0 (2.0, 33.0)	2.0 (1.0, 6.0)	20.0 (5.0, 42.5)	5.0 (1.0, 17.0)	2.0 (1.0, 4.0)	10.0 (4.0, 30.0)
Intra-articular injection (hyaluronic acid)	4.7 ± 3.88	1.5 ± 0.90	2.4 ± 1.91	4.0 ± 3.36	2.0 ± 1.41	2.8 ± 2.67
Median (IQR)	4.0 (2.0, 6.0)	1.0 (1.0, 2.0)	2.0 (1.0, 3.0)	3.0 (2.0, 5.0)	2.0 (1.0, 3.0)	2.0 (1.0, 3.0)
Intra-articular injection (steroids)	2.8 ± 2.82	1.1 ± 0.37	2.1 ± 2.95	2.1 ± 1.76	1.1 ± 0.47	1.6 ± 1.87
Median (IQR)	2.0 (1.0, 3.0)	1.0 (1.0, 1.0)	1.0 (1.0, 2.0)	1.0 (1.0, 2.0)	1.0 (1.0, 1.0)	1.0 (1.0, 2.0)
Opioids (weak)	43.1 ± 38.34	6.7 ± 4.45	47.6 ± 39.69	59.0 ± 40.55	5.9 ± 4.74	35.9 ± 37.13
Median (IQR)	30.0 (7.0, 74.0)	7.0 (4.0, 7.0)	41.5 (14.0, 72.0)	60.0 (22.0, 88.0)	6.0 (2.0, 7.0)	22.0 (7.0, 56.0)
Opioids (strong)	1.8 ± 1.68	2.1 ± 0.96	2.3 ± 1.46	2.1 ± 2.69	2.1 ± 0.89	2.3 ± 1.53
Median (IQR)	1.0 (1.0, 2.0)	2.0 (1.0, 3.0)	2.0 (1.5, 3.0)	1.0 (1.0, 2.0)	2.0 (2.0, 3.0)	2.0 (1.0, 3.0)
Antidepressants (SNRI)	53.1 ± 43.75	7.5 ± 5.32	62.1 ± 44.26	74.1 ± 40.81	7.4 ± 6.60	51.8 ± 41.30
Median (IQR)	35.0 (21.0, 96.0)	7.0 (6.0, 7.0)	47.5 (21.0, 90.0)	84.0 (42.0, 102.0)	7.0 (4.0, 7.0)	41.5 (14.0, 89.5)
Extract from inflamed cutaneous tissue of rabbits inoculated with vaccinia virus	45.8 ± 32.96	6.6 ± 1.27	40.9 ± 32.51	55.9 ± 36.28	6.9 ± 1.88	37.1 ± 26.52
Median (IQR)	35.0 (14.0, 84.0)	7.0 (6.0, 7.0)	30.0 (14.0, 59.0)	47.5 (29.0, 80.5)	7.0 (6.0, 8.0)	32.5 (14.0, 56.0)

*NSAID* non-steroidal anti-inflammatory drug, *OA* osteoarthritis, *IQR* interquartile range.

Pre-surgery period: from index date -91 days to index date -1 day; immediate post-surgery period: from index date to index date +6 days; post-surgery period: from index date +7 days to index date +89 days.

### 3.4 Factors affecting prescription of analgesics during the post-surgery period

Use of pre-operative oral NSAIDs (odds ratio [OR] 0.56, 95 % CI:0.44–0.72], p < 0.0001) and weak opioids (OR 0.58 [0.38–0.87], p = 0.0092) were associated with post-surgical use of analgesics in patients with hip OA. Pre-operative use of intra-articular hyaluronic acid was a significant factor in patients with knee OA (OR 0.45 [0.21–0.97], p = 0.0425) (Table 3). Certain comorbidities before TJR were associated with the prescription of analgesics in the post-surgery period: presence of gastritis and duodenitis (OR 0.59 [0.42–0.84], p = 0.0032), and mental disorders (OR 0.72 [0.53–0.97], p = 0.0289) in patients with hip OA (Table 3).

**Table 3:** Factors affecting the prescription of analgesics during the post-surgery period (knee/hip).

Factors	Knee OA		Hip OA	
	**Odds ratio (95 % CI)**	**P-value**	**Odds ratio (95 % CI)**	**P-value**
Age, 55–64 years (vs < 55 years)	1.54 (0.33–7.06	0.4022	0.96 (0.74–1.24)	0.2815
Age, ≥65 years (vs < 55 years)	1.08 (0.23–5.18)	0.7839	1.20 (0.83–1.75)	0.2351
Gender (vs male)	1.11 (0.51–2.42)	0.8000	1.05 (0.78–1.40)	0.7561
Presence of comorbidities				
Essential (primary) hypertension	1.02 (0.45–2.29)	0.9634	0.82 (0.61–1.12)	0.2169
Disorders of lipoprotein metabolism and other lipidemia	1.22 (0.53–2.81)	0.6448	0.89 (0.63–1.25)	0.5006
Sleep disorders	0.58 (0.22–1.49)	0.2577	1.07 (0.79–1.46)	0.6398
Gastritis and duodenitis	0.84 (0.34–2.10)	0.7116	0.59 (0.42–0.84)	0.0032
Other functional intestinal disorders	0.65 (0.24–1.76)	0.3974	1.14 (0.82–1.58)	0.4292
Iron deficiency anemia	1.11 (0.48–2.56)	0.8134	0.89 (0.70–1.14)	0.3705
Gastro-esophageal reflux disease	0.73 (0.28–1.87)	0.5084	–	–
Unspecified diabetes mellitus	0.53 (0.17–1.60)	0.2581	–	–
Phlebitis and thrombophlebitis	1.29 (0.52–3.16)	0.5841	1.07 (0.78–1.46)	0.6865
Spondylosis	1.12 (0.44–2.89)	0.8099	1.12 (0.82–1.53)	0.4676
Dorsalgia	–	–	0.92 (0.65–1.31)	0.6557
Presence of other functional implants	–	–	0.78 (0.55–1.11)	0.1669
Mental disorders	0.10 (0.44–2.27)	0.9978	0.72 (0.53–0.97)	0.0289
Duration ≥365 days (vs < 365 years)	1.15 (0.53–2.50)	0.7209	0.98 (0.77–1.25)	0.8841
Treatment (pre-surgery period)				
NSAID (oral)	0.69 (0.33–1.46)	0.3330	0.56 (0.44–0.72)	<.0001
NSAID (non-oral)	1.21 (0.56–2.62)	0.6300	0.80 (0.63–1.03)	0.0837
Acetaminophen	0.19 (0.02–1.53)	0.1193	0.76 (0.49–1.19)	0.2346
Intra-articular injection (hyaluronic acid)	0.45 (0.21–0.97)	0.0425	0.99 (0.55–1.79)	0.9720
Intra-articular injection (steroid)	1.77 (0.76–4.14)	0.1869	0.73 (0.41–1.28)	0.2702
Opioid (weak)	1.46 (0.60–3.54)	0.4008	0.58 (0.38–0.87)	0.0092
Opioid (strong)	3.68 (0.69–19.64)	0.1274	1.88 (0.73–4.80)	0.1896
Antidepressant (SNRI)	<0.0001 (<0.0001->999.9999)	0.9817	0.54 (0.16–1.78)	0.3079
Extract from inflamed cutaneous tissue of rabbits inoculated with vaccinia virus	2.29 (0.47–11.23)	0.3088	0.31 (0.07–1.32)	0.1129

*NSAID* Non-steroidal anti-inflammatory drug, *OA* osteoarthritis, *SNRI* serotonin-norepinephrine reuptake inhibitor.

### 3.5 Factors affecting prescription of opioids during the post-surgery period

Use of opioids before surgery was associated with post-operative use of opioids, thus affecting opioid prescription post-surgery: weak opioids for knee (OR 7.00 [4.65–10.54], p < 0.0001) and hip OA (OR 4.59 [3.44–6.13], p < 0.0001) and strong opioids for hip OA (OR 2.48 [1.01–6.07], p = 0.0468) (Table 4). Comorbidities positively associated with post-surgery use of opioids were the presence of mental disorders in knee OA (OR 1.49 [1.04–2.13], p = 0.0296) and functional intestinal disorders (OR 1.48 [1.07–2.05], p = 0.0178) and mental disorders (OR 1.51 [1.14–2.00], p = 0.0045) in hip OA. However, presence of other functional implants was negatively associated with the use of opioids post-surgery (OR 0.65 [0.43–0.99], p = 0.0425) in hip OA (Table 4).

**Table 4:** Factors influencing opioid prescription during the post-surgery period (knee/hip).

Factors	Knee OA		Hip OA	
	Odds ratio (95 % CI)	P-value	Odds ratio (95 % CI)	P-value
Age, 55–64 years (vs < 55 years)	0.71 (0.39–1.29)	0.3290	0.96 (0.72–1.27)	0.9408
Age, ≥65 years (vs < 55 years)	0.74 (0.40–1.36)	0.5158	0.90 (0.59–1.36)	0.6458
Gender (vs male)	0.82 (0.57–1.17)	0.2781	0.89(0.65–1.20)	0.4463
Presence of comorbidities				
Essential (primary) Hypertension	0.80 (0.55–1.16)	0.2384	1.10 (0.81–1.50)	0.5417
Disorders of lipoprotein metabolism and other lipidemia	0.77 (0.53–1.14)	0.1916	0.98 (0.69–1.39)	0.9066
Sleep disorders	0.98 (0.67–1.44)	0.9250	1.00 (0.73–1.37)	0.9798
Gastritis and duodenitis	0.96 (0.65–1.41)	0.8193	1.18 (0.86–1.61)	0.3112
Other functional intestinal disorders	1.46 (0.99–2.16)	0.0573	1.48 (1.07–2.05)	0.0178
Iron deficiency anemia	1.04 (0.72–1.52)	0.8236	0.77 (0.59–1.01)	0.0555
Gastro-esophageal reflux disease	1.26 (0.86–1.86)	0.2375	–	–
Unspecified diabetes mellitus	0.96 (0.63–1.46)	0.8400	–	–
Phlebitis and thrombophlebitis	1.14 (0.75–1.72)	0.5412	0.77 (0.53–1.11)	0.1574
Spondylosis	0.74 (0.48–1.16)	0.1910	0.83 (0.59–1.18)	0.3031
Mental disorders	1.49 (1.04–2.13)	0.0296	1.51(1.14–2.00)	0.0045
Dorsalgia	–	–	0.70(0.48–1.02)	0.0615
Presence of other functional implants	–	–	0.65 (0.43–0.99)	0.0425
Duration ≥365 days (vs < 365 years)	1.08(0.76–1.56)	0.6615	1.03(0.79–1.33)	0.8502
Treatment (pre-surgery period)				
NSAIDs (oral)	1.39 (0.98–1.97)	0.0649	1.15(0.87–1.51)	0.3183
NSAIDs (non-oral)	0.99 (0.69–1.42)	0.9556	1.01(0.78–1.32)	0.9252
Acetaminophen	0.96 (0.55–1.67)	0.8768	1.25(0.83–1.88)	0.2830
Intra-articular injection (hyaluronic acid)	0.83 (0.59–1.16)	0.2687	1.64(0.98–2.76)	0.0607
Intra-articular injection (steroid)	0.76 (0.50–1.16)	0.2021	0.73(0.43–1.26)	0.2619
Opioid (weak)	7.00 (4.65–10.54)	<0.0001	4.59(3.44–6.13)	<0.0001
Opioid (strong)	0.68 (0.22–2.12)	0.5109	2.48(1.01–6.07)	0.0468
Antidepressant (SNRI)	1.37 (0.54–3.47)	0.5026	0.80 (0.32–2.03)	0.6430
Extract from inflamed cutaneous tissue of rabbits inoculated with vaccinia virus	0.85 (0.36–2.00)	0.7061	1.09(0.47–2.54)	0.8346

*NSAID* Non-steroidal anti-inflammatory drug, *OA* osteoarthritis, *SNRI* serotonin-norepinephrine reuptake inhibitor.

## 4 Discussion

This is the first real-world study to report the difference in the use of analgesics before and after TJR, and to analyze the factors influencing the prescription patterns of these analgesics in patients with knee/hip OA in Japan. Our study showed that a significant number of patients were using analgesics even 3 months after TJR. NSAIDs were the most commonly used analgesics in the pre- and post-surgery periods, whereas strong opioids were the most common analgesics in the immediate post-surgery period in knee/hip OA patients. When compared with pre-surgery, prescription of analgesics, mainly NSAIDs, acetaminophen, intra-articular steroids, and opioids, increased in the immediate post-surgery period and reduced in the post-surgery period. Pre-operative use of oral NSAIDs, opioids, and intra-articular hyaluronic acid was associated with post-operative use of analgesics. Certain comorbidities, such as mental disorders and some gastrointestinal disorders, were associated with increased use of analgesics post-operatively.

In Japan, the prevalence of knee OA of Kellgren-Lawrence grade ≥2 (55.6 %) is higher than that of hip OA (1.4%–3.5 %), and more patients with knee OA undergo arthroplasties than those with hip OA.^15^^,^^16^ However, in this analysis, fewer patients underwent TKR versus THR. This observation could be attributable to two reasons. First, the JMDC database includes insurance claims for employees and their family members who are ≤75 years old, i.e., patients >75 years old are not included. Second, the etiology of hip OA is mostly congenital acetabular dysplasia, which is diagnosed at a younger age compared to knee OA, which is diagnosed at a relatively older age in Japan.^17^ The timing of a THR was earlier than that of knee OA in this study. The average age of patients undergoing joint surgery varies: most patients in their 50s and 60s undergo THR and those in their 70s undergo TKR. However, the average age of patients undergoing TKR was younger (∼64 years) in this study probably because the JMDC database includes only working age people and elderly people are not included. In general, the mean age of patients with knee OA was higher than that of patients with hip OA; this finding is consistent with actual clinical data.^10^^,^^18^ The proportion of comorbidities, such as hypertension, lipidemia, and diabetes, was higher in patients with knee OA compared with hip OA, and most patients with OA were women. Thus, the patient characteristics were similar to those reported in previous studies.^10^^,^^19^^,^^20^

The results of this study showed that compared to pre-surgery, the use of NSAIDs, acetaminophen, intra-articular steroids, and opioids increases in the immediate post-TJR period and reduces in the post-surgery period. As expected, the number of patients not using analgesics prior to surgery reduced immediately after surgery but the number increased in the post-surgery period. This finding suggests pain relief after 90 days of surgery; however, 96.1 % and 84.9 % of patients with knee OA and hip OA, respectively, still required analgesics to relieve their pain post-surgery. A similar trend of analgesics use was seen in a previous study.^21^ Moreover, NSAID use was predominant in the pre-surgery and post-surgery periods, whereas opioids were predominantly prescribed immediately post-surgery. In our study, higher use of analgesic drugs and strong opioids was reported immediately post-surgery, which may be due to the adaptation of multimodal approach for perioperative pain management comprising local anesthetic-based regional analgesic techniques combined with systemic opioids and other analgesics.^22^ These protocols are targeted to provide effective pain management immediately after surgery, as it is believed that these patients may perform well in rehabilitation and overall prognosis.^22^ The immediate post-operative period is often characterized by acute pain from surgery. Furthermore, it has been recognized recently that inadequate pain control at this stage may lead to prolonged post-operative pain. Surgery-induced pain is believed to disappear after the immediate post-operative period but a certain number of people continue experiencing pain.

Almost half of the patients with knee/hip OA who were discharged from the facilities but visited other facilities had received oral and non-oral NSAIDs. Moreover, in this category, the proportion of patients with knee OA was greater than that of hip OA, thus suggesting that patients with knee OA are more likely to have residual pain. In addition, post-operative satisfaction varies greatly, with many TJR patients experiencing chronic pain after surgery. Patient satisfaction was higher with THR than with TKR, and patients rarely refused to undergo THR.^23^^,^^24^ In our study, 20.7 % and 9.7 % of patients were not prescribed opioids before TJR but prescribed opioids after TJR in the knee and hip OA groups, respectively. This may reflect lower satisfaction in patients who underwent TKR. NSAIDs were the most common analgesics used in these patients, a finding in line with the results reported by previous studies and guidelines for OA pain management.^4^^,^^5^^,^^10^^,^^21^^,^^25^

Logistic regression analysis in our study showed association between pre-operative use of analgesics and post-operative prescription of these drugs. If a patient's pain becomes too severe before TJR, the patient is less likely to discontinue analgesics. Patients who received opioids before surgery may have experienced severe pain, and this is demonstrated by a significant association between pre-operative use of opioids and continued use of analgesics post-surgery in our study. Pre-operative use of opioids was a significant factor affecting post-operative opioid use in a previous study.^26^ It is noteworthy to mention that opioids are hard to discontinue and might be used after surgery continuously. Therefore, opioids should be cautiously used in OA to avoid concerns about drug dependence.

Patients with comorbid mental disorders could use antidepressants and other medications. There have been reports of new-onset depression being associated with pre-operative comorbid anxiety and pre-operative, post-operative, and ongoing opioid use after TJR. Therefore, additional caution is advised while using opioids in these patients.^27^^,^^28^ Moreover, another safety concern arises from the reported association between NSAID use and cardiovascular (CV) adverse events in patients with OA.^29^ Given that approximately 50 % of patients in our study had CV comorbidity, and 60%–80 % of knee OA patients and 60%–70 % of hip OA patients were using oral NSAIDs, these patients are at a high risk for developing CV adverse events. Therefore, precautionary monitoring for CV events in these patients should be considered.

A major strength of this analysis is that data are extracted from a large-scale claims database in Japan and allows patients to be tracked even when they are transferred to other hospitals. Post-operative prescription of analgesic drugs after discharge from facilities to other facilities (such as general practitioners) included in this JMDC analysis aids understanding of the current situation in Japan. Nonetheless, there are certain limitations of the study that need to be considered. Prescription status of concomitant medications was not examined in this analysis but could be researched further. There is nonavailability of medical chart information, data of the elderly (≥75 years), and disease severity in the database. Information on functional outcome of patients who underwent knee/hip TJR is not available in this database; hence, correlation between analgesic use and functional outcomes could not be determined. Moreover, the reasons for analgesic choices could not be assessed. Generally in Japan, analgesic choice is made by the treating physician based on the overall condition of the patient, or sometimes, analgesics are used in accordance with the pain management protocol of the clinical facility. The database used in this study does not include the reasons for prescribing analgesics; therefore, it is difficult to confirm these observations. There was a higher number of prescriptions for intra-articular hyaluronic acid after surgery observed in this study, which could be attributed to its use to treat OA at non-index joints or for non-OA conditions. As it is highly important to prevent infection after artificial joint surgery, intra-articular injections are generally not performed after surgery. In addition, there was no information regarding the prescription of over-the-counter drugs.

This real-world study reports the differences in analgesic use before and after TJR in patients with knee/hip OA in Japan. A significant number of patients were using analgesics even after 3 months of TJR. Use of strong opioids also increased. Pre-operative analgesic use associated with continued use post-surgery, indicating that efficient and optimal pain management before and immediately after TJR may be important to reduce post-operative use of analgesics, especially opioids that pose a risk of drug dependence.

## Conflict of interest

NE, HY, and KT are employees of 10.13039/100010793Pfizer Japan Inc., and hold stock/stock options of Pfizer Inc.; TT is an employee of Clinical Study Support, Inc., and was involved in the study based on their contract with 10.13039/100010793Pfizer Japan Inc.; TS and MD were not financially compensated for their collaboration in this project nor for the development of this manuscript; however, TS and MD have received honorariums from Pfizer Japan Inc. outside of this work. The authors report no other conflicts of interest in this work.

## Funding

This work was supported by 10.13039/100010793Pfizer Japan Inc. Editorial and medical writing support and publication charges were funded by 10.13039/100010793Pfizer Japan Inc.
