# Supplementary material for: Use of analgesics before and after total joint replacement in working-age Japanese patients with knee and hip osteoarthritis: A retrospective database study
Source: Asia Pac J Sports Med Arthrosc Rehabil Technol. 2023 Nov 3;35:1–8. doi: 10.1016/j.asmart.2023.10.002 (PMC10659993; doi:10.1016/j.asmart.2023.10.002)
Supplement: Multimedia component 1 [file mmc1.docx]

**Table S1 [file format .docx]:** Analgesics used in the post-surgery period for patients discharged from hospitals where they were operated for TJR but post-procedure analgesic use was recorded at another hospital site

| Parameters | Knee OA  N= 356 | Hip OA  N= 677 |
| --- | --- | --- |
| Analgesics, n (%) |  |  |
| Oral NSAID | 196 (55.1) | 357 (52.7) |
| Non-oral NSAID | 218 (61.2) | 325 (48.0) |
| Acetaminophen | 52 (14.6) | 94 (13.9) |
| Intra-articular hyaluronic acid injection | 54 (15.2) | 51 (7.5) |
| Intra-articular steroid injection | 44 (12.4) | 96 (14.2) |
| Mild/weak opioid | 44 (12.4) | 53 (7.8) |
| Strong opioid | 4 (1.1) | 12 (1.8) |
| Antidepressant (SNRI) | 7 (2.0) | 11 (1.6) |
| Cutaneous tissue extract* | 6 (1.7) | 8 (1.2) |
| Prescription (pain medication) days, mean (SD) |  |  |
| Oral NSAID | 33.5 (31.62) | 26.4 (24.39) |
| Non-oral NSAID | 3.1 (5.32) | 2.1 (3.02) |
| Acetaminophen | 12.2 (13.93) | 10.2 (17.32) |
| Intra-articular hyaluronic acid injection | 2.7 (2.07) | 2.8 (2.58) |
| Intra-articular steroids injection | 2.1 (3.52) | 1.6 (1.10) |
| Mild/weak opioid | 39.0 (30.69) | 29.9 (25.69) |
| Strong opioid | 2.5 (1.29) | 2.8 (3.24) |
| Antidepressant (SNRI) | 70.6 (28.04) | 41.9 (37.30) |
| Cutaneous tissue extract* | 36.8 (29.42) | 26.0 (18.35) |
| Number of drug used per patient (oral and non-oral NSAIDs were counted separately), mean (SD) | 1.4 (0.71) | 1.3 (0.62) |
| Number of drug types (oral and non-oral NSAIDs were counted as single class/drug), mean (SD) | 1.8 (0.90) | 1.5 (0.79) |

*NSAID* nonsteroidal anti-inflammatory drug, *OA* osteoarthritis, *SNRI* serotonin-norepinephrine reuptake inhibitor.

*Extract from inflamed cutaneous tissue of rabbits inoculated with vaccinia viru
